# Effect of Hexagonal Boron Nitrides Injection on the Survival of Dorsal Pedicle Skin Flap in Rats: An Experimental Study

**DOI:** 10.3390/nano16010029

**Published:** 2025-12-24

**Authors:** Fatma Nilay Tutak, Ozan Balik, Ebru Annac, Azimet Ozdemir, Semra Bulbuloglu

**Affiliations:** 1 Vetal Animal Health Products Joint Stock Company, 02040 Adiyaman, Turkey; nilayprs@gmail.com; 2 Clinic of Plastic Reconstructive and Aesthetic Surgery by Ozan Balık, 34330 Istanbul, Turkey; ozan.balik@gmail.com; 3 Department of Histology and Embryology, Faculty of Medicine, Adiyaman University, 02040 Adiyaman, Turkey; eelibol@adiyaman.edu.tr; 4 Clinic of Plastic Reconstructive and Aesthetic Surgery by Azimet Ozdemir, 34371 Istanbul, Turkey; dr.aziozdemir@gmail.com; 5 Division of Surgical Nursing, Nursing Department, Faculty of Health Sciences, Florya Campus, Istanbul Aydin University, 34295 Istanbul, Turkey; 6 Department of Midwifery, Faculty of Health Sciences, Istanbul Aydin University, 34295 Istanbul, Turkey

**Keywords:** hexagonal boron nitride, pedicled dorsal flap, flap survival, distal necrosis, pedicled flap

## Abstract

**Background:** Dorsal pedicle skin flap application is a cover procedure frequently used by plastic surgeons to cover acute and chronic wounds, but preventing postoperative flap loss and disruption of the wound healing process has not yet been achieved. Injecting boron nitride during the transfer of the dorsal pedicle skin flap may increase flap survival. **Objective:** This study investigated the efficacy of hexagonal boron nitride (hBN) injection in enhancing the survival of pedicled skin flaps harvested from the dorsal region of rats. **Method:** This study employed an experimental design. A total of 24 Wistar albino rats were divided into three groups of eight each: Control (Group 1), Sham (Group 2), and Experimental (Group 3). A 27 cm^2^ (3 cm × 9 cm) dorsal skin flap with a proximal pedicle was harvested at the level of the iliac crests, with the flap extending cranially, and then reattached. During flap transfer, no intervention was performed in Group 1, physiological saline was injected into Group 2, and hBN was injected into Group 3. After a certain period of time, sections were taken from the proximal pedicle skin flap on the dorsal side of the rats, and histochemical examination and biochemical analyses were performed on these sections. **Results:** In this study, it was observed that the epithelial integrity of the epidermal layer was disrupted and the epithelium was thinned in places in Group 2. Compared to Group 1, collagen fiber density was lower, collagen fiber arrangement was irregular, and mast cell density was higher. In Group 3, similar to Group 1, the epidermis and dermis layers were composed of multilayered flat keratinized epithelium, collagen fiber density was high and had a regular arrangement, and elastic fiber structure was of normal density. The TGF-β1 and MMP-1 measurement results for the three groups were compared, and no statistically significant difference was found between the groups (*p* > 0.05). **Conclusions:** The results of this study support the benefit of hBN injection in improving flap survival after proximal pedicle skin flap application on the dorsal side of rats. Although the improved healing of skin layers after flap transfer with hBN suggests that it supports cell proliferation, the mechanism of action and pathophysiology remain unclear.

## 1. Introduction

The dorsal pedicled skin flap has been in the literature since 1965 [1], but it is linked to a bunch of complications, like dryness, infection, and partial necrosis of the recipient site in the donor area [2]. These complications continue to pose significant limitations in the use of the dorsal pedicled skin flap [3,4]. A previous study reported that using a new flap (15,673 cm^2^ + SD3.37) smaller than the dorsal pedicled skin flap (18,904 cm^2^ + SD3.79) was associated with fewer complications [5]. After the dorsal pedicle skin flap is transferred to the recipient site, good neo/revascularization is desired, as this is expected to ensure flap survival. To this end, numerous treatments have been developed to improve flap physiology and support neo/revascularization. Some of these treatments include capsaicin ointment [6,7], flashlamp-pumped pulsed-dye laser [8,9], and the combination of nonivamide and nicoboxil [10].

Despite the application of therapeutic interventions aimed at increasing the survival of dorsal pedicle skin flaps, the development of complications cannot be completely prevented. Boron, a trace element that supports wound healing by inhibiting pro-inflammatory cytokines and reducing the inflammatory response [11], has attracted the attention of researchers. Boron is obtained through dried fruits, red wine, and certain vegetables [12]. Among its known effects are increasing bone density, supporting wound healing, and enhancing liver function [13]. These positive properties suggest that boron may also be explored for its potential in treating diseases. In this context, it has been used for bone and skin issues due to its anti-inflammatory effects and is beneficial [14,15]. A previous study emphasized that boron is an effective method for repairing impaired neovascularization in acute and chronic wounds [16].

Studies using various boron-containing components (GPBT2 hydrogel, Cu-BIF/CS membrane, bioactive silicon–boron-containing gels) have reported that wound healing is completed with an increase of up to 30% in the formation of the epithelial layer [16,17,18,19]. Boron is known to have significant effects, including increasing fibroblast activity and angiogenesis, inducing M2 macrophage polarization and subsequent cytokine release, reducing scarring, and enhancing wound healing [18]. Recent studies have shown that BN helps filter water contaminated with antibiotics, organic compounds, and nitro compounds [20], can be used in cancer therapy [21], and enhances bone tissue regeneration in osteoblasts [22]. Dorsal pedicle skin flaps pose a significant clinical challenge in terms of infection and necrosis development. BN, which possesses various biological functions, is thought to be effective in enhancing flap survival. BN is a thermally and chemically refractory compound of boron and nitrogen. In this study, we aimed to investigate the efficacy of hBN in ensuring the survival of dorsal pedicle skin flaps.

## 2. Materials and Methods

### 2.1. Research Design and Rat Selection

This study is an experimental in vivo research. The sample of the study consisted of 24 male Wistar albino rats weighing approximately 200–215 g. The 24 rats were divided into three groups of eight each: Control, Sham, and Experimental. Each rat was placed in a separate cage, and a 12 h light-dark cycle was applied. All rats were fed ad libitum. Histopathological evaluation was performed in the laboratories of the Department of Histology and Embryology, Faculty of Medicine, Adıyaman University.

### 2.2. Procedure

**Scheme 1:**
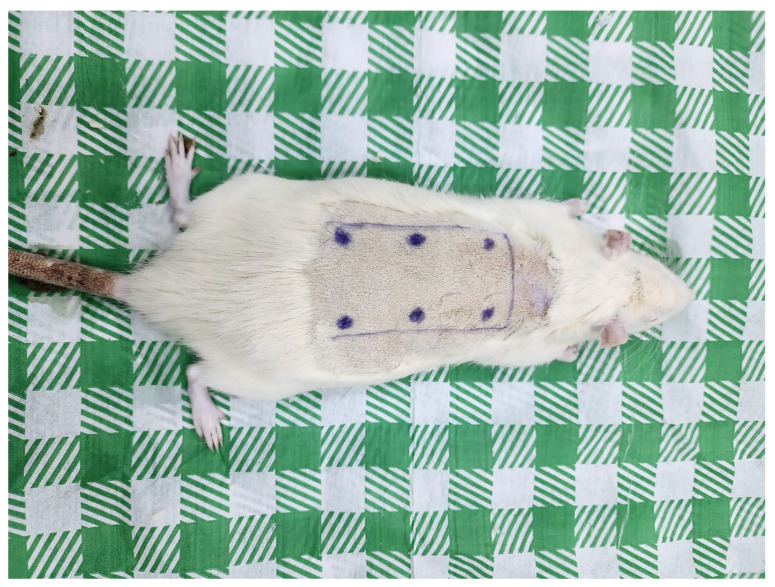
Harvesting of Dorsal Pedicled Skin Flap Followed by Injection of Physiological Serum and hBNs into 6 Marked Points.

In our study, intraperitoneal anesthesia was administered to all rats, divided into three groups during the procedure. For this purpose, 5 mg/kg Xylazine and 50 mg/kg Ketamine were injected. The rats were placed in the prone position, the hair on their backs was shaved, and the area was disinfected with povidone-iodine solution. In the surgical plan for each rat, the upper edges of the iliac crests and the spinal line (spine course) were identified, and the dorsal pedicled skin flap was marked. In all rats, a standard pedicled skin flap measuring 27 cm^2^ (3 cm × 9 cm) was planned to be harvested at the level of the iliac crests, with both sides 1 cm away from the spine, and the flap extending cranially. In all groups, the flap was harvested to include the panniculus carnosus proximally and was immediately sutured back to its original position with 4/0 Prolene without any further manipulation. No intervention other than surgery was performed on the rats in Group 1 (control). The six green dots shown in Scheme 1 were marked. In Group 2 (sham group), 0.2 mL (total 1.2 mL) of physiological saline was injected into each of the 6 marked points. In Group 3 (experimental group), 0.2 mL/0.2 mg (total 1.2 mL/1.2 mg) of hBN was injected into each of the 6 marked points. Scheme 1 shows the dorsal pedicled skin flap after harvest, with the 6 marked points where physiological serum and hBN were injected. 72 h after the surgical procedure, the back skin was shaved with an electric razor, and tissue samples were dissected from the dorsal pedicle skin flap for histopathological and biochemical analysis [23], and the rats were sacrificed. To prevent the rats from self-mutilating their flaps, each was fitted with an Elizabethan collar.

### 2.3. hBNs Nanoparticles

hBN nanoparticles have properties similar to carbon nanotubes and carry boron and nitrogen atoms instead of carbon nanotubes. hBN does not occur naturally, so it is synthesized artificially from boron and nitrogen precursors to obtain structures similar to graphene [24]. When hBNs are synthesized, boron and nitrogen atoms are arranged in a hexagonal structure and bond covalently on top of each other. Like graphene, two-dimensional (2D) hBN layers interact with each other through van der Waals forces [25], and form spherical nanoparticles [26].

### 2.4. hBNs Synthesis

A total of 2 g of boron sources (boric acid, boron trioxide, and colemanite) and 3 mL (13.38 M) of ammonia solution (nitrogen) were dissolved [24,27]. The resulting solution was transferred to a silicon carbide tray and left to dry on a heating plate at 100 °C for 20 min. After drying, the silicon carbide tray was placed in a Protherm PTF 14/50/450 furnace and exposed to a flow of ammonia gas for 2 h, heated to 1300 °C at 10 °C/min. Then, the silicon carbide tray was removed from the furnace at approximately 540–550 °C. In the final stage, hBN was scraped off the surface of the silicon carbide tray with a spatula. The obtained hBN was placed in vials, and 1 mg of hBN was diluted with 1 cc of distilled water.

### 2.5. SEM and TEM Imaging

SEM and TEM were used to characterize hBNs morphology and size (Helios Nano-Lab 600i FIB/SEM, FEI). SEM (Helios Nano-Lab 600i FIB/SEM, FEI) imaging was performed by sputtering at 60 nA/25 s using 3 nm thick conductive layer samples. TEM images were obtained from a JEOL-2100 HRTEM microscopy system at 200 kV (LaB6 filament/the Oxford Instruments 6498 EDS system).

### 2.6. Histopathological Examination

Skin samples were fixed in a 10% formaldehyde solution for one week, then routinely histologically examined using an automatic tissue tracking device (Leica TP1020, Nussloch, Germany) and embedded in paraffin blocks. Sections 5 μm thick were obtained from the paraffin blocks using a Thermo Shandon Finesse ME microtome (Thermo Fisher Scientific, Cheshire, UK). The sections were stained with hematoxylin-eosin, Masson’s trichrome, toluidine blue, and Verhoeff stains for histopathological examination. Evaluations were performed using a Carl Zeiss Axiocam ERc5 model (Carl Zeiss Microscopy GmbH 07745, Jena, Germany) digital camera-equipped microscope.

### 2.7. Biochemical Analyses

The TGFBR1-Cat.No E1148Ra kit was used in biochemical analyses. The analysis kit was produced using the enzyme-linked immunosorbent assay (ELISA) method. The plate was pre-coated with rat TGFBR1 antibody. TGFBR1 present in the sample is added and binds to the antibodies coated on the wells. Then, biotinylated rat TGFBR1 antibody is added and binds to TGFBR1 in the sample. Streptavidin-HRP is added and binds to the biotinylated TGFBR1 antibody. After incubation, unbound streptavidin-HRP is washed away during the washing step. Then, substrate solution is added, and color develops in proportion to the amount of TGFBR1 in the rat. The reaction is terminated by adding an acidic stop solution, and absorbance is measured at 450 nm.

The second ELISA-linked kit is MMP-1-Cat.No E0312Ra. The plate is pre-coated with Rat MMP-1 antibody. MMP-1 present in the sample is added and binds to the antibodies coated on the wells. Then, biotinylated Rat MMP-1 Antibody is added and binds to MMP-1 in the sample. Streptavidin-HRP is added and binds to the biotinylated MMP-1 antibody. After incubation, unbound Streptavidin-HRP is washed away during the washing step. Then, the substrate solution is added, and color develops in proportion to the amount of rat MMP-1. The reaction is terminated by adding an acidic stop solution, and the absorbance is measured at 450 nm.

### 2.8. Data Analysis

The variables included in the study were described using descriptive statistics such as frequency, percentage, arithmetic mean, standard deviation, confidence intervals, and graphs. The Shapiro–Wilk normality test was used to determine whether the data were normally distributed. For data that were normally distributed, the parametric test, One-way ANOVA, was used for comparison. The significance level upper limit was set at *p* = 0.05 and the confidence interval at 95%. The data were analyzed using the SPSS 27.0 for Windows software package.

## 3. Findings

**Figure 1:**
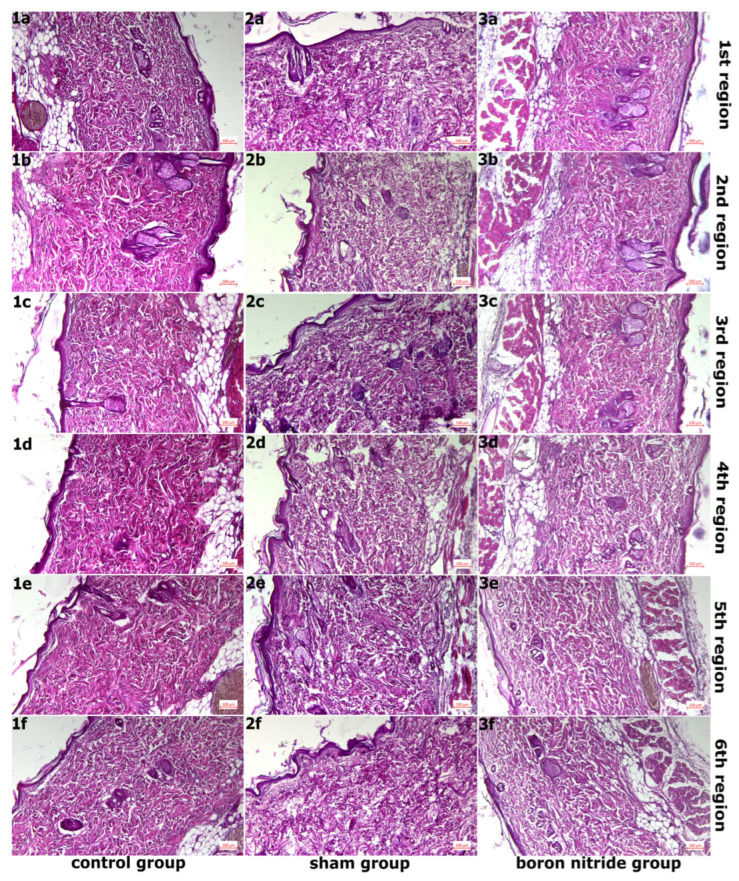
Images Obtained After ×10 Objective Magnification of Sections Taken From All Groups After H&E Staining. (**1a**–**1f**,**2a**–**2f**,**3a**–**3f**) In the control group, healthy tissue was observed, while in the sham group, there was an increase in collagen fiber structures with disruption of normal organization. In the hBN group, normal tissue organization was preserved compared to the sham group.

**Figure 2:**
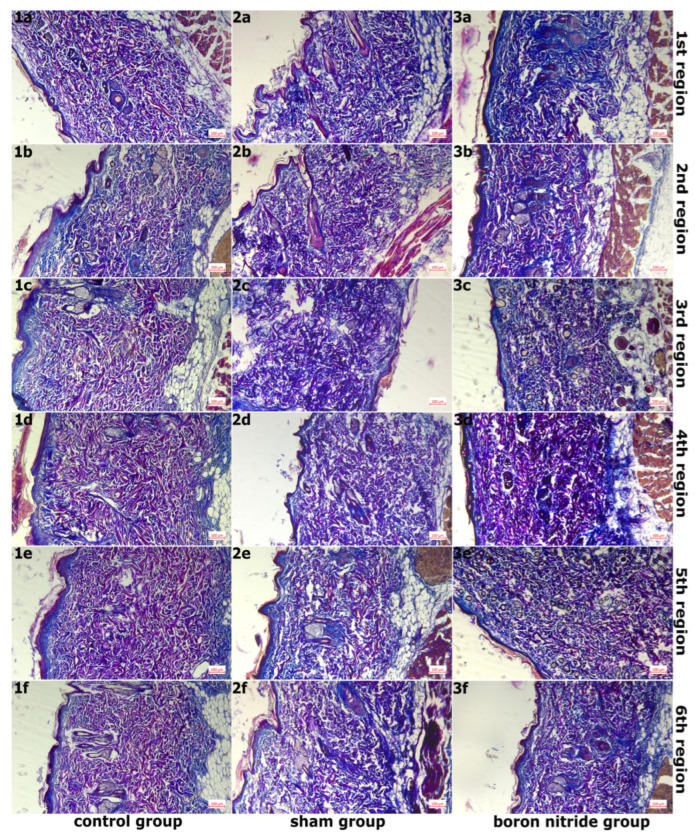
Images of Sections Taken From Groups After Masson Triple Staining, Magnified 10 Times With an ×10 Objective Lens. (**1a**–**1f**,**2a**–**2f**,**3a**–**3f**) In the control group, collagen fiber density was normal, while in the sham group, collagen fiber density decreased, and connective tissue edema was observed. In the hBN group, normal tissue organization was preserved compared to the sham group.

**Figure 3:**
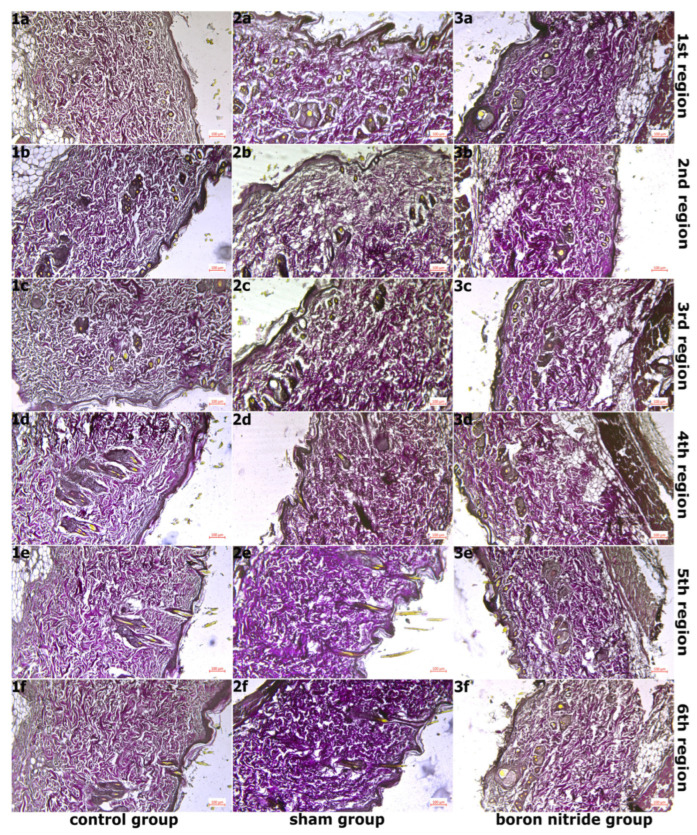
Images of Sections Taken from Groups After Verhoeff–Van Gieson Staining at 10× Magnification. (**1a**–**1f**,**2a**–**2f**,**3a**–**3f**) In the control group, elastic fibers were observed to have normal density and normal organization, while in the sham group, elastic fibers were observed to have disrupted normal organization. In the hBN group, normal tissue organization was observed to be preserved when compared to the sham group.

**Figure 4:**
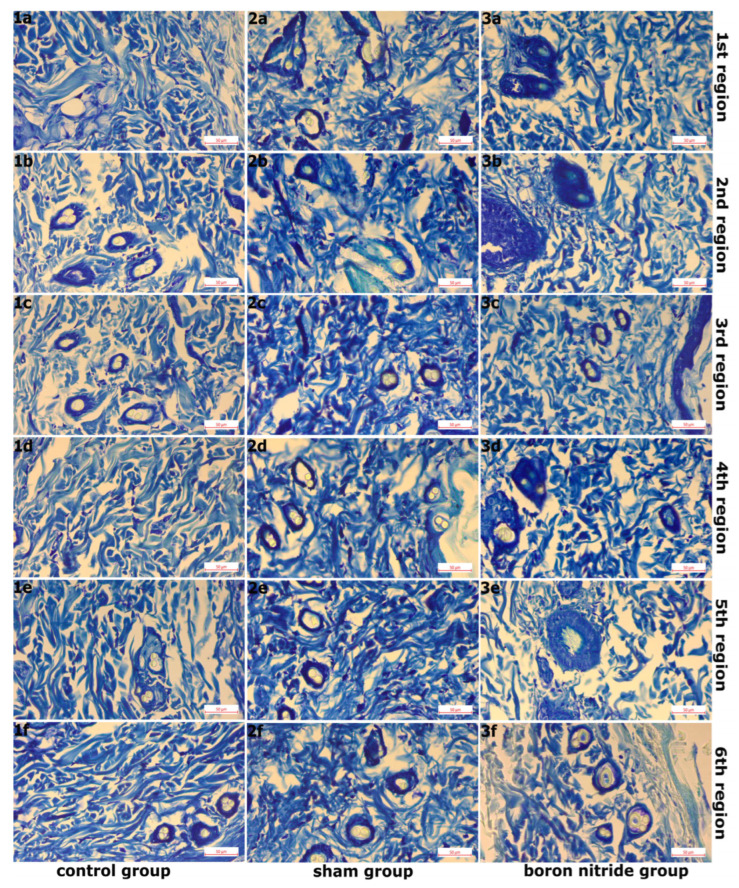
Images of Sections Taken from Groups After Toluidine Blue Staining and Magnified with a 40× Objective Lens. (**1a**–**1f**,**2a**–**2f**,**3a**–**3f**) Normal mast cell density in the control group, increased mast cell density in the sham group, and normal mast cell density in the hBN group.

Figure 1 shows images obtained after ×10 objective magnification of tissue sections taken from all groups after H&E staining. In the examination of the 6 marked areas belonging to Group 1, the epidermis and dermis layers appeared normal and healthy. The epidermis consisted of a multi-layered flat keratinized type of epithelium. The dermis layer had a rich structure of normally organized collagen fiber structures. These were accompanied by connective tissue cells and blood vessels (Figure 1(1a–1f) and Figure 2(1a–1f)). In addition, normal density elastic fiber structure was observed (Figure 3(1a–1f)). In the connective tissue, mast cells were present at normal density between the fibers (Figure 4(1a–1f)).

Figure 2 shows images of sections taken from the groups after Masson’s trichrome staining, magnified 10 times. In the examination of six areas in the sham group, it was observed that the integrity of the epithelial structure belonging to the epidermis layer was disrupted in some areas, and the epithelium was thinned in places. A slight decrease in collagen fiber density compared to the control group and disruption of the normal organization of collagen fibers were detected. Edema was observed in the connective tissue (Figure 1(2a–2f) and Figure 2(2a–2f)). In addition, elastic fiber structure was observed at normal density (Figure 3(2a–2f)). Mast cell density between fibers in the connective tissue was increased compared to the control group (Figure 4(2a–2f)).

Figure 3 shows images of sections taken from the groups after Verhoeff–Van Gieson staining at ×10 objective magnification. In the examination of the 6 regions in hBN group, a similar image to the control group was observed in the areas of regions 1 and 2. The epidermis and dermis layers had a normal, healthy appearance. The epidermis consisted of a multi-layered, flat, keratinized epithelium. The dermis layer had a rich structure composed of normally organized collagen fiber structures. These were accompanied by connective tissue cells and blood vessels. (Figure 1(3a,3b) and Figure 2(3a,3b)). Additionally, elastic fiber structures of normal density were observed (Figure 3(3a,3b)). Connective tissue edema was observed in the areas belonging to regions 3 and 4. Furthermore, when compared to the sham group, collagen and elastic fiber structures were observed to be better organized in these regions (Figure 1(3c,3d), Figure 2(3c,3d) and Figure 3(3c,3d)). Connective tissue edema was observed to be increased in the areas belonging to regions 5 and 6 compared to other regions. Furthermore, when compared to the sham group, collagen and elastic fiber structures were observed to be better organized in these regions (Figure 1(3e,3f), Figure 2(3e,3f) and Figure 3(3e,3f)). Figure 4 shows images of sections taken from the groups after Toluidine blue staining and magnified 40×. In all regions, mast cells were present at normal density between the fibers in the connective tissue (Figure 4(3a–3f)). It was determined that hBN application preserves normal tissue structure compared to the sham group.

No signs of hemorrhage or inflammation were observed in any of the regions of all groups in the histopathological examination.

Table 1 includes biochemical parameter results of tissues belonging to groups. The mean values of TGF-beta receptor type-1 (TGF-β1) (ng/mL) in the control, sham, and hBN group were 8.99 ± 1.83, 11.55 ± 0.76, and 10.54 ± 0.26, respectively. and no statistically significant difference was detected among the three groups (*p* = 0.543). The mean values of Metalloproteinase-1 (MMP-1) (ng/mL) were 3.74 ± 1.52, 4.71 ± 1.10, and 4.69 ± 1.05, respectively, and no statistically significant difference was detected among the three groups (*p* = 0.749).

## 4. Discussion

The 24 rats in the sample of this study were divided into three groups: Group 1 (control), Group 2 (sham), and Group 3 (hBN). During flap transfer, 0.2 mL of physiological saline or hBN was injected into six marked points in the rats in Groups 2 and 3. After waiting 72 h, histopathological scoring and biochemical analysis were performed on sections taken from the flap. Skin flaps are frequently used by plastic surgeons to compensate for tissue loss following cancer surgery, for wound repair, and for the treatment of ulcers. A skin flap is a piece of tissue with its vascularity that is harvested from the donor site and transferred to the recipient site by sliding it over [28]. This study was an in vivo investigation conducted on rats, and the harvested dorsal pedicled skin flap was reattached to the donor site.

Distal necrosis development and reduced apoptosis following ischemic reperfusion after flap transfer are significant risk factors threatening flap survival. To neutralize these risk factors, prostaglandin synthesis inhibitors, alpha-adrenergic antagonists, and topical nitrates that support vascularity appear to be effective solutions [29,30]. Additionally, the deactivation of proinflammatory cytokines, stem cell therapies, and certain treatments have been reported to prevent ischemic reperfusion [31,32,33]. In previous studies, the use of dorsal pedicled skin flaps in rats was similar to the measurements in this study, at 27 cm^2^ (3 cm × 9 cm) [34,35,36,37]. A previous study emphasized that reducing the dorsal pedicled skin flap by one-third resulted in fewer complications [5]. Necrosis development after flap transfer is characterized by tissue appearance with eschar formation and darkened skin color on the postoperative zero or first day. In this study, tissue sections were taken 72 h after flap transfer. In Group 2 (Sham), abnormalities and thinning of the epithelial structures of the epidermis were observed in some areas. Additionally, the density of collagen fibers was reduced, and their structure was irregular. The group that received hBN (Group 3) exhibited dermis and epidermis characteristics similar to the control group, with collagen fiber density and structure at a good level. hBN injection eliminated the risk of distal flap necrosis and ischemic reperfusion in dorsal pedicle skin flaps. Additionally, it strengthened the keratinization property of the epidermis by supporting keratinocyte production.

The expression of free oxygen radicals, Tumor Necrosis Factor-α (TNF-α), Inhibitor of kappa B (IκB) nuclear factor kappa B (NF-κB), and Interleukin (IL)-6 results in tissue damage, leading to ischemic reperfusion [38], which in turn leads to the development of distal necrosis. Oxidative stress, reactive oxygen species, and disruption in angiogenesis and extracellular matrix (ECM) formation trigger partial flap loss, leading to adverse esthetic and functional outcomes [39,40]. A previous study demonstrated that deferoxamine use improved microvascular density and flap survival by increasing HIF-1α and VEGF expression [41]. A previous study reported that boron plays a regulatory role in the aforementioned chemicals [42]. It has been noted that MMP-1 production increases in pathological tissues, whereas TGF-β1 inhibits cytokine-induced MMP-1 gene expression in fibroblasts. Thanks to the inhibitory effect of TGF-β1, the induction of NF-κB-specific gene transcription is eliminated [43]. In this study, there was no statistically significant difference in the average values of TGF-β1 and MMP-1 among the three groups, but the average values of TGF-β1 and MMP-1 were highest in the sham group. This finding may provide clues that hBN injection prevents uncontrolled tissue destruction.

Ischemia–reperfusion injury, microcirculation failure, increased inflammation, poor tissue repair, and distal necrosis result in decreased flap viability [44,45]. When skin flaps are harvested from areas distant from central veins, distal necrosis may be triggered; however, angiogenesis is very important in increasing survival after skin flap transfer. Angiogenesis is defined as the process of new vessel formation from pre-existing vasculature that provides adequate nutrient and oxygen transport [46], and an increase in microvascular density observed under a microscope indicates good vasculature. hBN, one of the subtypes of BN, is a nanostructure formed by B and N atoms arranged in a hexagonal network resembling graphene. hBN has been reported to improve cancer by reducing apoptotic death caused by reactive oxygen species and metastasis through its activation within cells [47]. A study comparing hBN with boric acid reported that hBN improved angiogenesis by increasing cell proliferation and migration and that its antioxidant capacity was higher than that of boric acid, thereby reducing apoptosis [48]. The hBN nanoparticles used in this study were approximately 200–300 nm in size. Rats in Group 3 received 0.2 mL/0.2 (total 1.2 mL) of hBN injections at six separate sites.

Boron is known to have various effects on wound healing. Oral and topical boron use has been reported to increase leukocyte density in rats [49] and reduce excessive inflammation by inhibiting proteases [50]. In this study, mast cell density increased in Group 3, which received hBN injections, similar to the literature, and there was no increase in inflammation. Boron has been used in the treatment of various diseases in the past. These diseases include vaginitis [51], malignancy [12,14], and skin and bone (osteoporosis and osteoarthritis) diseases [14,15]. Additionally, boron has been beneficial in the treatment of wounds, burns, and various skin ulcerations [52,53]. BN is thought to modulate inflammation and angiogenicity by enhancing fibroblast organization, collagen activity, antimicrobial resistance, and pro-angiogenic signaling [54,55]. In a previous study, a randomized dorsal flap model was created in rats, and histological and proteomic changes were evaluated. The same study found that boron-based topical treatment significantly improved flap survival, increased granulation tissue formation, promoted organized collagen deposition, and reduced inflammatory infiltration [56]. To our knowledge, this study is the first to examine the efficacy of hBN injection in increasing the survival of dorsal pedicled skin flaps. The results of this study demonstrated that hBN reduced tissue destruction, increased cell proliferation and mast cell density, and maintained the normal balance of inflammation.

## 5. Limitations

This study has several limitations. The study is an in vivo study and cannot be generalized to human subjects. It is unknown whether the total amount of hBN applied during flap transfer (1.2 mL/1.2 mg) is sufficient. The effect of hBN on TNF-α and interleukins, free oxygen radicals, or other unstudied chemical products (TNF-α, IL-6, VEGF, CD31 and other oxidative stress markers) remains unknown. Furthermore, the histopathological analysis was purely qualitative. Quantitative data such as collagen fiber density (using ImageJ), microvascular density (CD31 staining), and mast cell count could not be provided. Biochemical assays were limited. Key factors related to flap ischemia–reperfusion injury (e.g., TNF-α, IL-6, SOD, MDA) and angiogenesis (e.g., VEGF) could not be detected to clarify the underlying mechanism of BN. Macroscopic evaluation of flap survival was not provided. Data on tissue sections obtained from phlebs are limited to 72 h post-surgery and cannot be generalized to longer-term outcomes.

## 6. Conclusions

In this study, the sample consisted of 24 rats divided into groups of eight. In all groups, the dorsal pedicled skin flap was harvested from each rat and then transferred back to the donor site. No intervention was performed on Group 1, while physiological serum was injected into the marked areas on the skin flap in Group 2, and hBN was injected in Group 3. In Group 3, where hBN was applied, the flap appearance was similar to that of the control group (Group 1) in the sections taken 72 h after the surgical intervention. hBN injection helped maintain the integrity of the epithelial structure by preserving the boundaries between the epidermis and dermis layers and contributed to the formation of a multilayered squamous keratinized epithelium in the epidermis by supporting keratinocyte production. Additionally, hBN injection increased collagen fiber structure, elastic fiber structure, and mast cell density. According to the results of this study, hBN did not increase hemorrhage or inflammation after dorsal pedicled skin flap transfer. hBN injection had no association with TGF-β1 and MMP-1 release. The results of this study are expected to guide future studies. In this context, hBN may be beneficial in improving the survival of dorsal pedicled skin flaps.

## Figures and Tables

**Table 1:** Biochemical Parameter Results of Tissues Belonging to Groups.

Group	Control	Sham	Bor	Test and Sig.
TGF-beta receptor type-1 (TGF-β1 ng/mL)	8.99 ± 1.83	11.55 ± 0.76	10.54 ± 0.26	F = 1.283, *p* = 0.543
Metalloproteinase-1 (MMP-1 ng/mL)	3.74 ± 1.52	4.71 ± 1.10	4.69 ± 1.05	F = 1.721, *p* = 0.749

F = One-way analysis of variance—ANOVA.

## Data Availability

The datasets generated and/or analyzed in this study are available from the corresponding author upon reasonable request.
